# Utilizing Big Data for Public Health

**DOI:** 10.2188/jea.JE20160036

**Published:** 2016-03-05

**Authors:** Hisatomi Arima

**Affiliations:** Center for Epidemiologic Research in Asia, Shiga University of Medical Science, Otsu, Shiga, Japan

Big data is usually defined as information assets characterized by such high volume, velocity, and variety that specific technology and analytical methods are required for its transformation into value.^1^ Analysis of big data may provide useful information in a variety of areas, including healthcare.^2^ Numbers of data sets obtained from routine operations in the area of healthcare have rapidly increased in recent years, due to cheap and advanced information collection systems, but challenges in extracting useful information from large and complex datasets remain.

In this context, an accompanying article published in this issue of the *Journal of Epidemiology* investigated outcomes after out-of-hospital cardiac arrest using data from the All-Japan Utstein registry for the Fire and Disaster Management Agency, which is a nationwide population-based registry based on guidelines for uniform reporting of cardiac arrest.^3^ The proportion of patients in the registry with favorable neurological outcomes was 1.6% during the national meetings of the Japanese Society of Intensive Care Medicine, the Japanese Association for Acute Medicine, and the Japanese Circulation Society, while the proportion was 1.5% during the remaining days of the year. Likewise, 1-month survival rates were comparable between meeting days (3.8%) and non-meeting days (3.8%). The present analysis of big data from the nationwide registry of cardiac arrests supports the robustness of the emergency healthcare system in Japan and suggests that the quality of emergency care is maintained even when many professionals in emergency medicine are absent from their hospitals to attend medical conferences.

As demonstrated in the accompanying article,^3^ big data may provide useful information enabling the public sector and healthcare providers to assess their healthcare systems and distribution of resources. Big healthcare data also has great potential to improve our understanding of the effectiveness of treatments in the real world, as well as of the incidence, management, and prognosis of various medical conditions, particularly for rare diseases. Useful information obtained from big data will allow health professionals to provide better medical care. Although the privacy of individuals’ information must be protected, considering the great potential of big data, healthcare data generated within governmental processes should be open to researchers who have unique ideas on providing useful information regarding public health.
